# Supplementary figures and images for: Multi-scale modelling of location- and frequency-dependent synaptic plasticity induced by repetitive magnetic stimulation in the dendrites of pyramidal neurons
Source: PLoS Comput Biol. 2025 Nov 25;21(11):e1012295. doi: 10.1371/journal.pcbi.1012295 (PMC12671772; doi:10.1371/journal.pcbi.1012295)

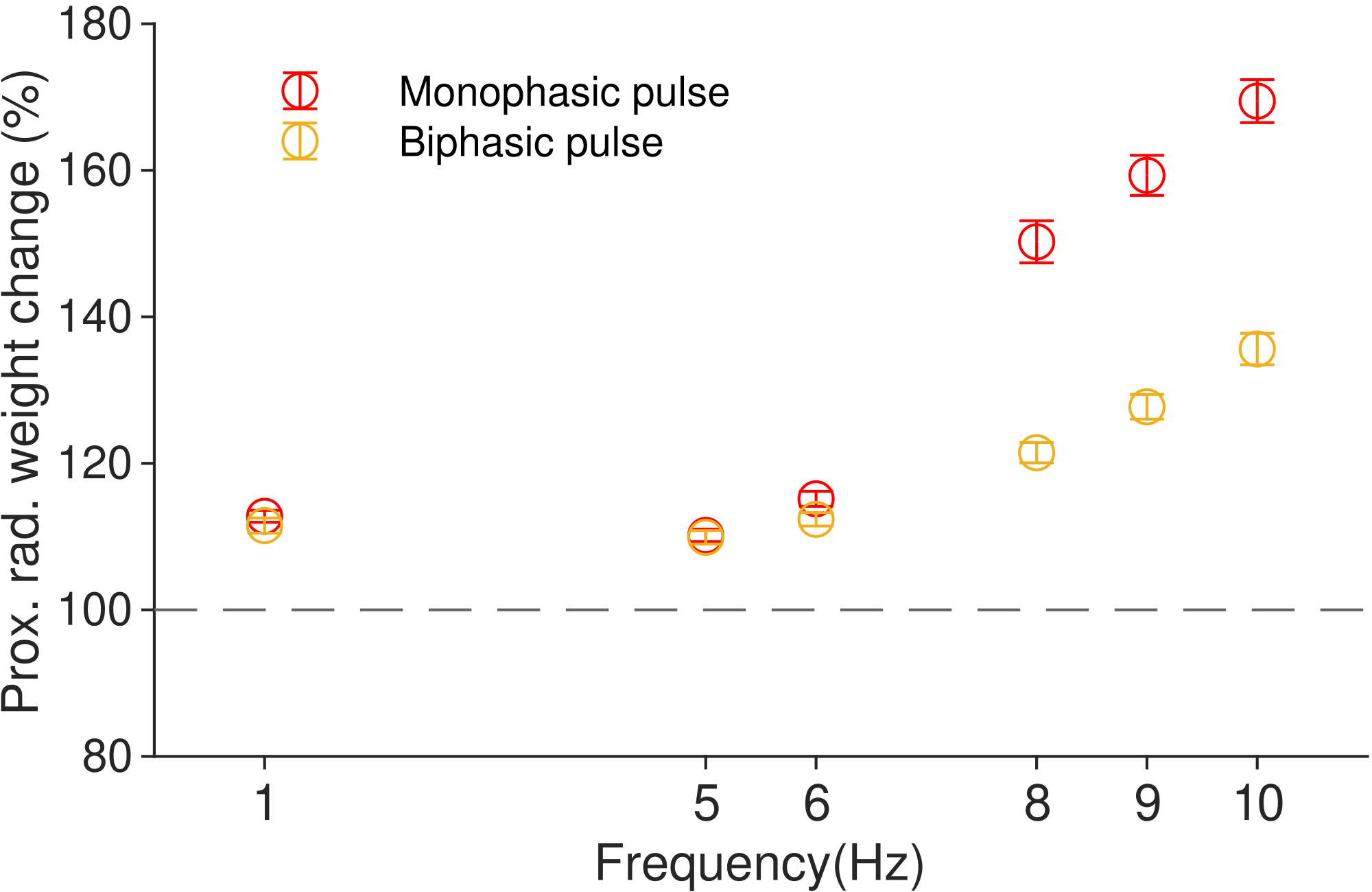

Supplement: S1 Fig — Biphasic stimulus produces much less LTP than monophasic stimulus for 9 and 10 Hz cases, but there is no noticeable difference for lower frequencies. (TIFF) [file pcbi.1012295.s001.tif]

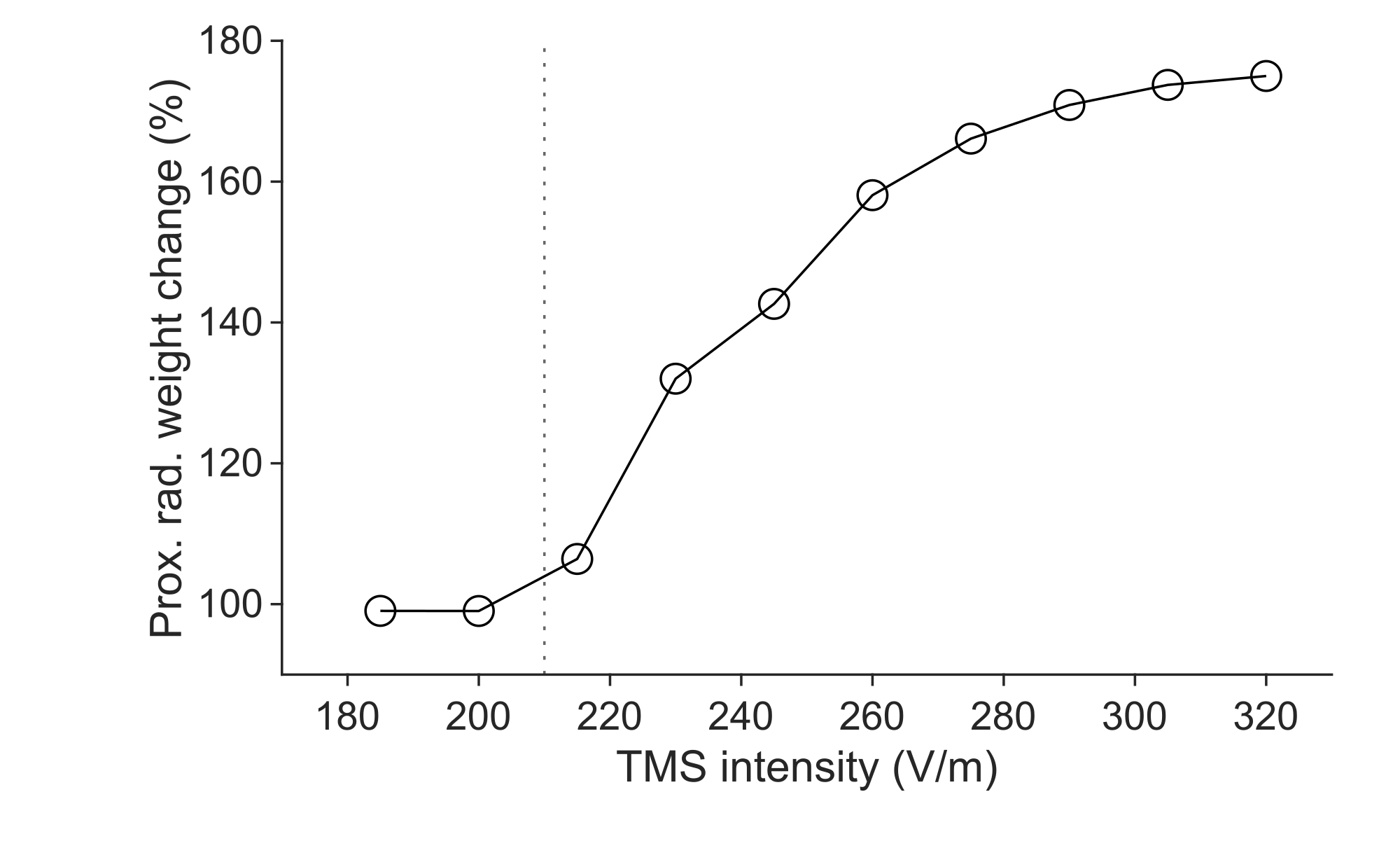

Supplement: S2 Fig — Below the firing threshold of 210 V/m, no LTP is observed, whereas once over the firing threshold, LTP amplitude increases, plateauing with intensities over 300 V/m. (TIFF) [file pcbi.1012295.s002.tif]

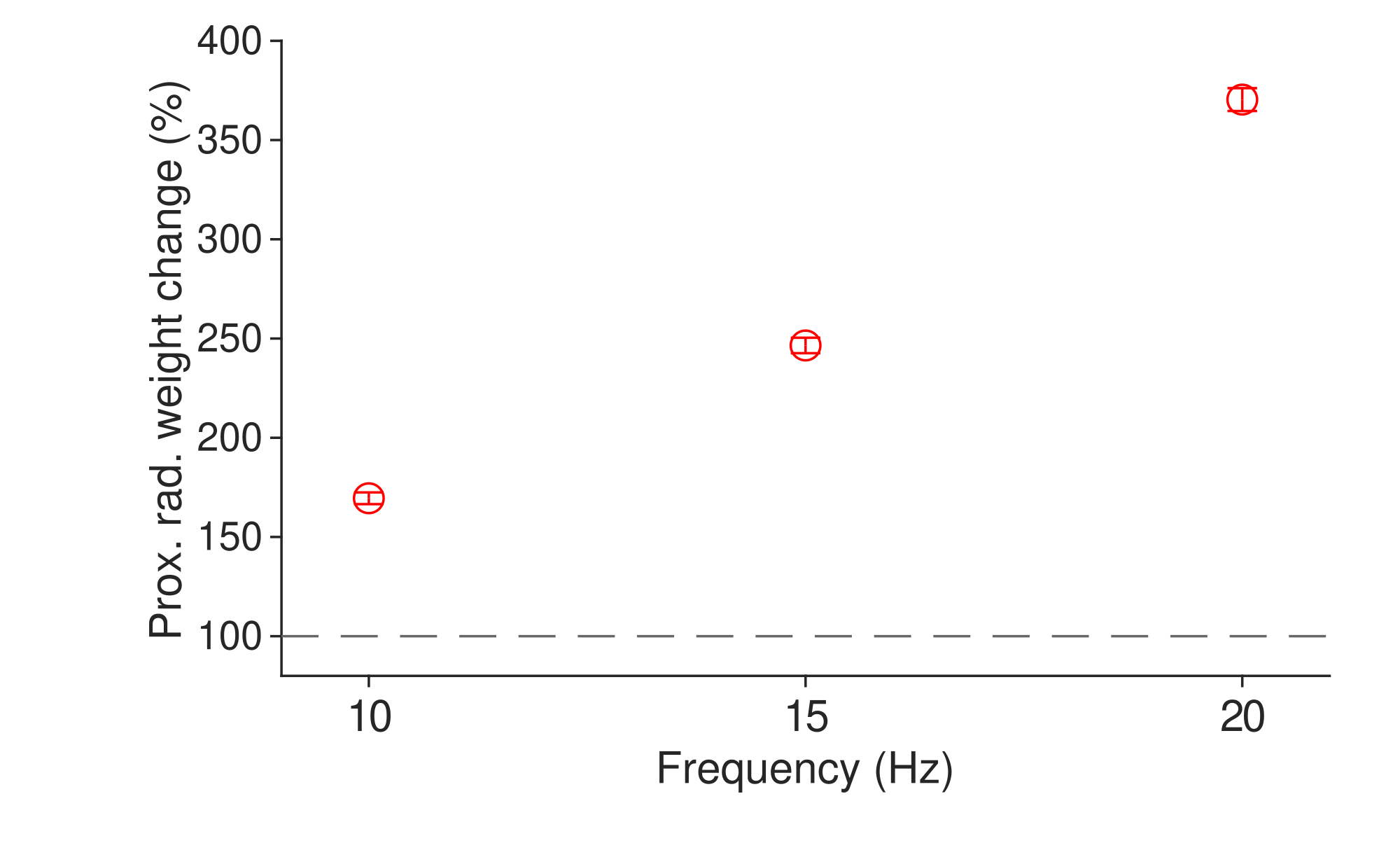

Supplement: S3 Fig — At higher frequencies than 10 Hz, proximal str. radiatum weights increase by greater amounts, in a linear relationship with increasing frequency. (TIFF) [file pcbi.1012295.s003.tif]

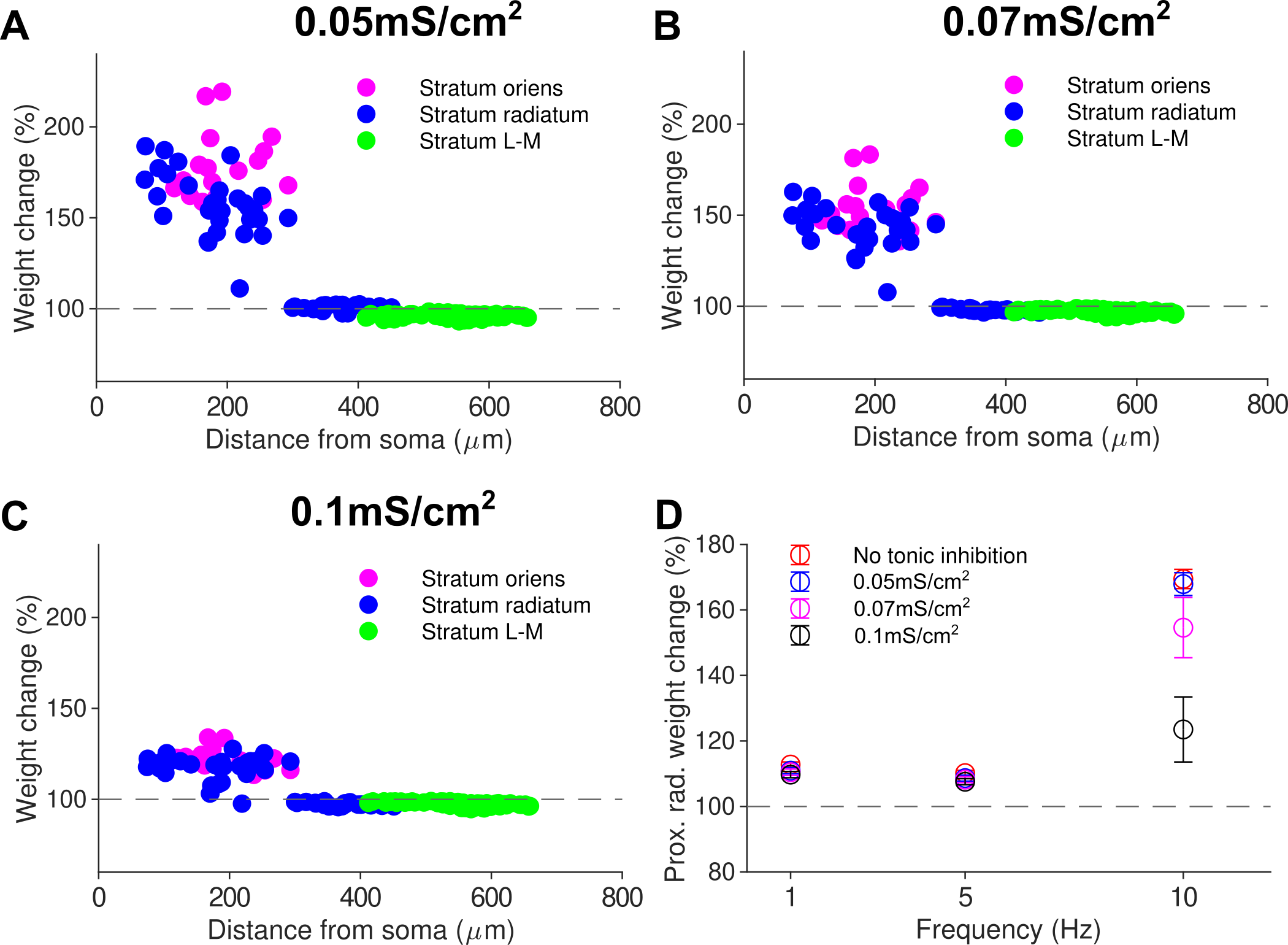

Supplement: S4 Fig — Effect of tonic inhibition on distance and frequency dependence of long-term potentiation. (a–c): Induction of LTP with 10 Hz 900 pulse protocol with varying tonic inhibition conductances (0.5–1 x 10^−4^ S/cm2, corresponding to 20–40 pA). (d) Frequency dependence of proximal LTP induction with different tonic inhibition conductances from variable frequency 900 pulse protocol. (TIFF) [file pcbi.1012295.s004.tif]

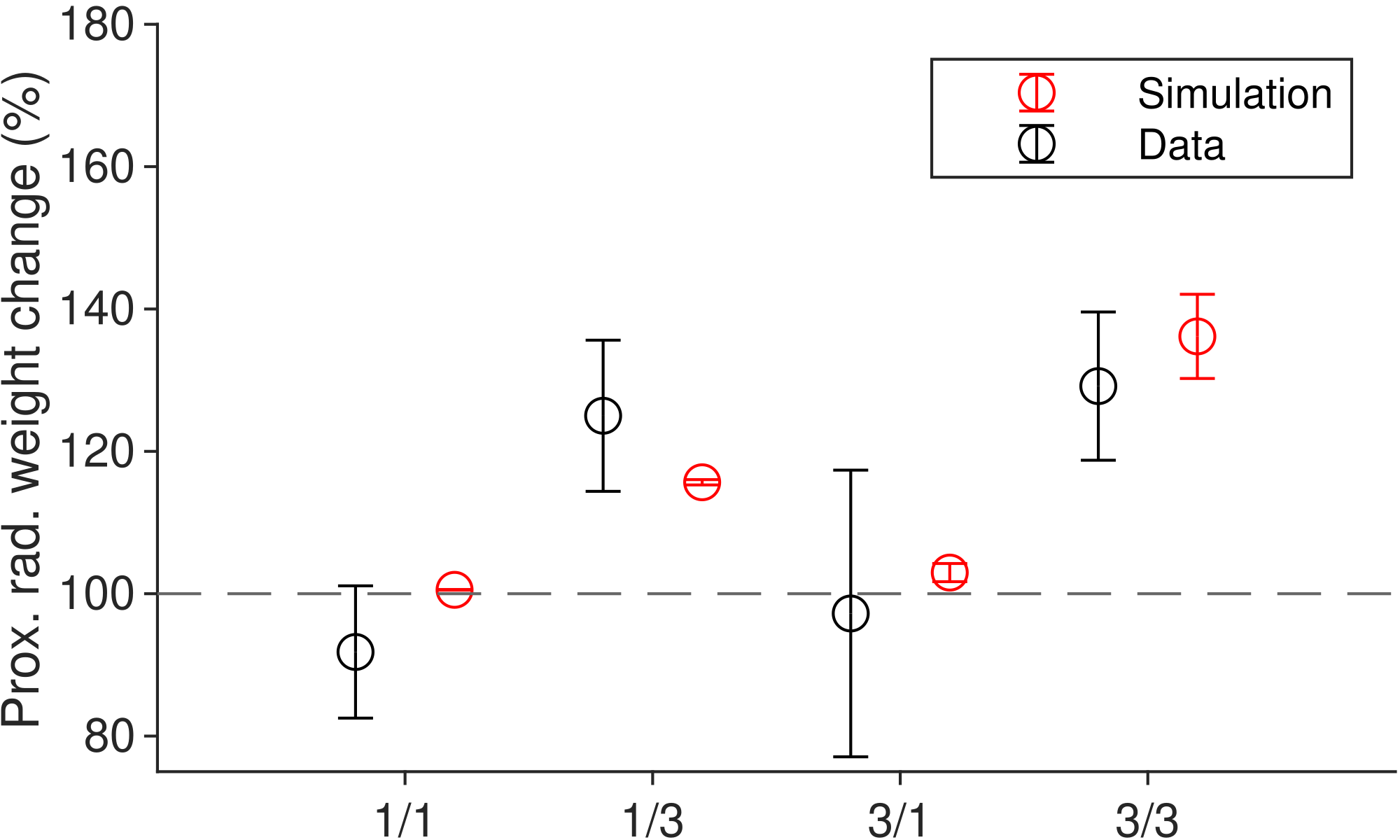

Supplement: S5 Fig — LTP is not induced by single paired postsynaptic stimulus (cases 1/1 and 3/1 for single presynaptic or 3x presynaptic paired with 1x postsynaptic) but is produced by burst postsynaptic stimulus (cases 1/3 and 3/3 for single or 3x presynaptic paired with 3x postsynaptic). (TIFF) [file pcbi.1012295.s005.tif]

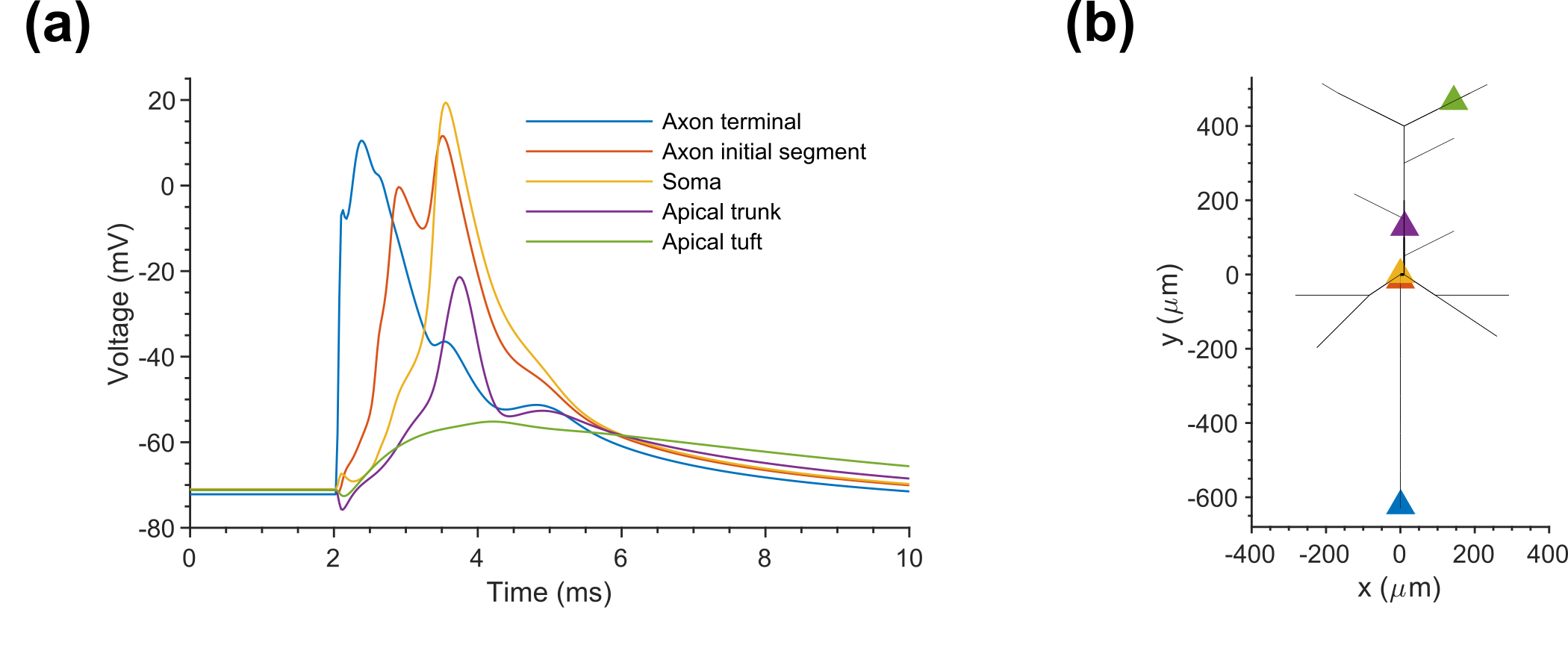

Supplement: S6 Fig — Propagation of an action potential induced by a 250 V/m monophasic TMS pulse. (a): Voltage trace at various recording locations showing initiation of action potential at axon terminal (b): Schematic of cell showing the recording locations. (TIFF) [file pcbi.1012295.s006.tif]

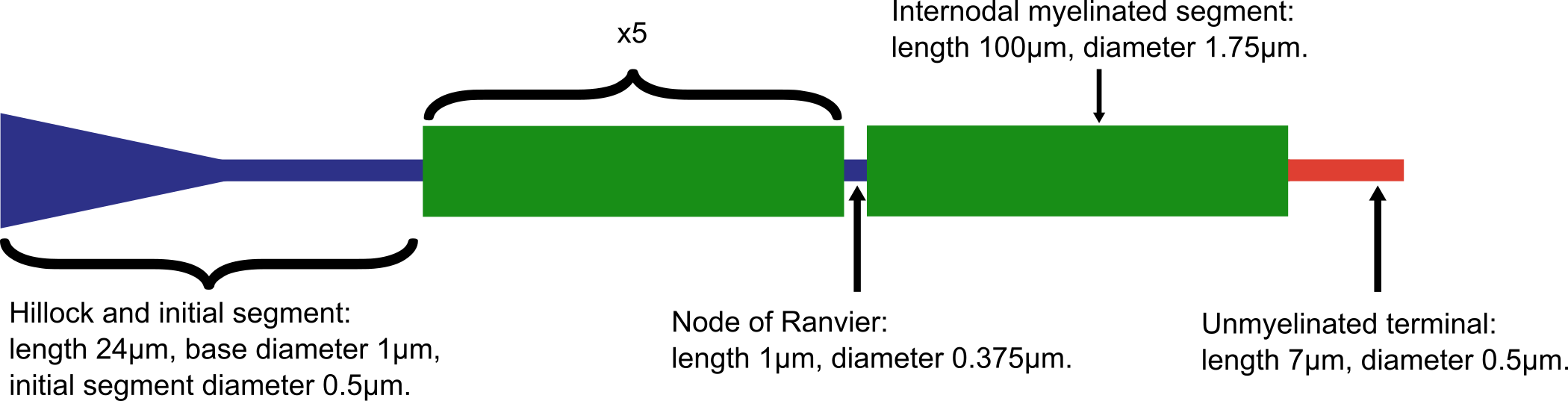

Supplement: S7 Fig — Same color segments have identical biophysics. Blue: Axon initial segment and nodes; Green: Myelin; Red: Unmyelinated terminal. (TIFF) [file pcbi.1012295.s007.tif]

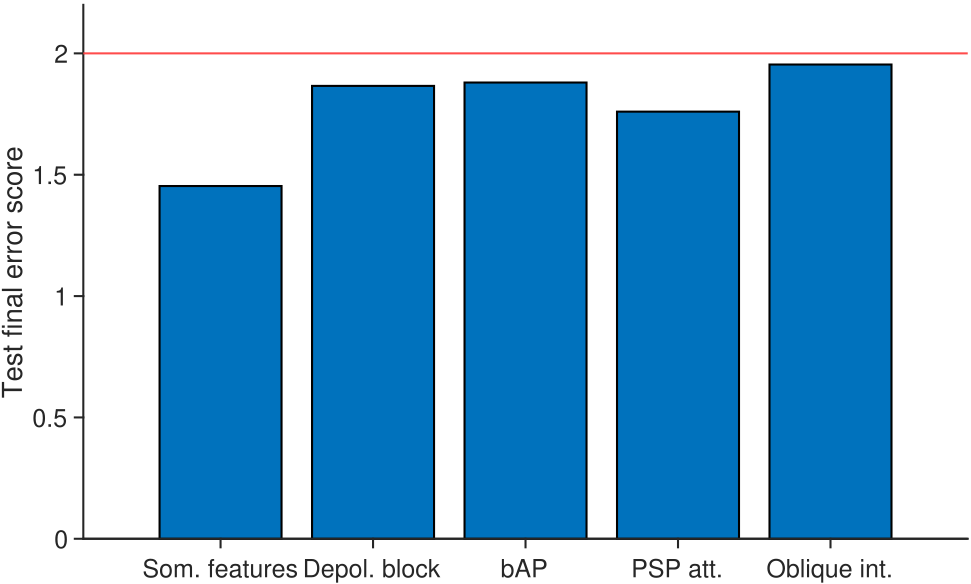

Supplement: S8 Fig — Error scores from HippoUnit test suite for 1. somatic spiking features, 2. depolarisation block, 3. back- propagating action potentials (bAPs), 4. attenuation of EPSPs, 5. nonlinear synaptic integration in oblique branches. Red line represents the acceptability threshold of two standard deviations. The model performed within two standard deviations of the experimental data and was considered acceptable. (TIFF) [file pcbi.1012295.s008.tif]
