# Supplementary material for: Multi-scale modelling of location- and frequency-dependent synaptic plasticity induced by repetitive magnetic stimulation in the dendrites of pyramidal neurons
Source: PLoS Comput Biol. 2025 Nov 25;21(11):e1012295. doi: 10.1371/journal.pcbi.1012295 (PMC12671772; doi:10.1371/journal.pcbi.1012295)
Supplement: Attachment — Submitted filename: Hananeia_et_al___Rebuttal_letter.pdf [file pcbi.1012295.s009.pdf]

JUSTUS-LIEBIG-UNIVERSITÄT GIESSEN

Ernst Strüngmann Institute
in Cooperation with Max Planck Society

FIAS Frankfurt Institute for Advanced Studies

PLoS Computational Biology, May 10, 2025
Cambridge, UK

Dear editors of *PLoS Computational Biology*,

We would like to submit the attached revised manuscript entitled ”Multi-scale modelling of location- and frequency-dependent synaptic plasticity induced by repetitive magnetic stimulation in the dendrites of pyramidal neurons” for publication as a journal paper in PLoS Computational Biology. We found the reviewers' comments very helpful and we feel that we have been able to thoroughly address their comments and improve the manuscript. We made figure updates, created new figures and tables and amended the text of the manuscript. We provide a detailed response to the reviewers' individual comments below.

If the reviewers have any further comments, we would be happy to address them, especially as we had to complete the final revision changes quickly because the PLoS Computational Biology staff could not automatically extend the revision deadline beyond 29 April. The revisions were done thoroughly but the tight deadline meant that the manuscript might still improve with a few additional finishing touches. The delay in the revision was due to the fact that the first author was in the middle of an international relocation (which was only completed last week by resolving immigration issues). Also, the revision required us to run a number of time consuming simulations.

We hope that the revised manuscript will be acceptable for publication in PLoS Computational Biology.

We look forward to hearing from you,

Nicholas Hananeia, Christian Ebner, Christos Galanis, Hermann Cuntz, Alexander Opitz, Andreas Vlachos & Peter Jedlicka

Reviewer 1

- **Reviewer 1 major issue 1: The manuscript reads like a draft that has not been edited or even ready by any of the senior authors. One can find many weird expressions (such as NMDAR supression: one inhibits a receptor/channel or supresses its activity) and jargon in the manuscript. The writing feels sloppy — there are entire paragraphs (e.g. lines 49-57, 111-122) that are completely incomprehensible.**

  We have rewritten substantial parts of the manuscript for clarity. See the improved text in the Introduction on p. 4-5.

- **Reviewer 1 major issue 2: The link to the model allows only to download a part of it. The reviewer is unable to test if the code runs on their machine.**

  We have adjusted our submitted code for usability, tested it with third-party testers, and provided detailed instructions for how to generate the simulation set. See the link: `https://www.dropbox.com/scl/fo/31hxt5ckf980u3f7exoqf/ACgS-I-AHtFrM8v75V87OX8` `rlkey=x8hyjd1lg8tenrigadrfu1maz&st=m3o6ful0&dl=0` For the publication, the code will be shared via `https://zenodo.org`.

- **Reviewer 1 major issue 3: The methods section is insufficient. The model neuron is not sufficiently described. The dataset it has been fitted to is not described. The synaptic plasticity model is not described at all. There are many instances of including technical details that were not described in the methods section e.g. line 134 a model variable N alpha that the reader knows is set to 0. It is also impossible to look at the model code to check oneself what are introduced changes.**

  We have addressed the concerns raised by providing a detailed description of the specific models and experimental protocols employed, as well as a clear explanation of the relevant parameters in the Methods section (see new Table 1 and lines 90-103 and lines 140-153). For the description of wellness of data fit for the cell model, we direct the reviewer to Supplementary Figure S8, and Subsection "Validation of the CA1 pyramidal cell model using HippoUnit tests" of Methods on Page 8.

- **Reviewer 1 major issue 4: There is no justification provided for using a model of pyramidal CA1 neuron fitted to slice data to replicate experiments with organotypic hippocampal cultures. There is no discussion on the validity and limitations of this approach.**

  We have added discussion about the choice of the pyramidal CA1 model and the limitations thereof. See Discussion p. 29-30. In addition, we emphasize in Methods that the choice of the pyramidal CA1 model has been validated by its performance in 5 different HippoUnit tests, see p. 8

- **Reviewer 1 major issue 5: Tonic inhibition. In addition to inhibitory synapses (phasic inhibition) there are also extrasynaptic GABAA receptors that excert a strong in-**

hibitory influence, usually reducing cell's excitability. The dataset used to fitting utilized mice older than 3 weeks with ACSF not including GABAAR inhibitors (not blocking tonic GABA inhibition, which is developing/present), so the model neuron implicitly includes this tonic inhibition. In validation of the synaptic plasticity model using Ikegaya et al data, which was collected in 19–25 days old rats. Some of these rats would not have developed tonic inhibition. The in-silico experiments should address the question of higher excitability of neurons in Ikegaya et al. data. Blocking inhibition in TBS simulations should address the issue of tonic inhibition.

We have followed the suggestion of the reviewer and implemented tonic inhibition (as used in our previous paper - Kolbaev et al. 2020) at various intensities in the realistic range for a CA1 pyramidal cell (Caraiscos et al. 2004), and demonstrated that our key findings of distance-dependent plasticity (i.e. proximal LTP) and frequency dependence thereof are robust even with the influence of tonic inhibition present; see new Supplementary Figure 3 and text on p. 20, lines 338-350.

- **Reviewer 1 major issue 6: Please use more than one synaptic plasticity paradigm in addition to data from synaptic plasticity experiments utilizing organotypic slice cultures to fit and validate the synaptic plasticity model.**

  In line with the reviewer's suggestion, we have expanded our simulation results by including an additional synaptic plasticity paradigm/dataset from hippocampal CA1 acute slice recordings in young adult rats (Pike et al. J Physiol 1999), which our model successfully replicates (see new Supplementary Figure 4). Furthermore, the plasticity model used in this study has been extensively validated in previous work, accurately capturing a range of observed phenomena, including spike-timing-dependent plasticity (STDP) induced by various pairing protocols, dendritic spike-induced plasticity, and subthreshold event-induced plasticity, as demonstrated in Figures 2,3,4,and 5 of Ebner et al. (2019). We mention it now on p. 13 and p. 16, lns. 283-287.

- **Reviewer 1 major issue 7: Please include plots with measurable variables, so it is easier to compare model predictions with experimental data.**

  We have included measured data points of observed synaptic weight changes to Figure 2, 3B and from pharmacological perturbation experiments to our updated Figure 5.

Reviewer 1 minor issues:

- **The title refers to modeling transcranial magnetic stimulation (TMS), which is actually not modeled in the manuscript. The authors reproduce in silico experiments with organotypic hippocampal cultures using magnetic stimulation. It is not a manuscript on modeling TMS.**

  We have renamed the manuscript's title (Now "Multi-scale modelling of location- and frequency-dependent synaptic plasticity induced by repetitive magnetic stimulation in

the dendrites of pyramidal neurons") and the nomenclature inside the article to refer to *repetitive magnetic stimulation (rMS)*, wherever appropriate.

- **Matlab is propriety, hence the in-silico experiment is not reproducible.**

  Although we agree that Python-based models would be more accessible, Matlab is widely used in the computational neuroscience community, and most academic institutions have purchased licenses. Hence, we believe that its wide availability in the community makes these tools easy to use and the experiment reproducible. In the future we would like to create a Python version of the NemoTMS.

- **I would personally move all the tables from the supplement to the methods section to make it easier to read.** We have followed this suggestion. It has been done in Tables 1 and 2.

- **Fig2: Ikegaya et al. data were collected in slice. There might be a good reason for adding noise, but it is not reproducing the non silent nature of slice cultures.** We have tried to use a plausible frequency of background activity, which is lower than in vivo frequencies (8 Hz) used in our previous modelling (Jedlicka et al. Plos CB 2015). We would like to mention that in slices too a substantial spontaneous activity can be present (Mao et al. Neuron 2001).

- **There definitely is a better way to evaluate the fit than the statement in line 216. Why are there no errorbars for simulated data (line 76 states that there were 10 test models).** Thank you, errorbars have been added to Figure 2 and RMSE has been used to quantify the goodness of fit., see lines 283-287.

- **line 74, 92 test battery→ test suite** This has been corrected and now reads "test suite". See p. 7 and8, l. 113 and 127 and Supplementary Material p. 6 and 7.

- **line 102 Exp2Syn is probably a double exponential. Provide mathematical description.** This has been corrected and described as a double exponential. See p. 8, l. 138

- **line 168 I do not know what "behaved within acceptable electrophysiological limits for a CA1 pyramidal cell" means. Please provide a figure illustrating that.** This referred to the CA1 pyramidal cell model producing acceptable results within two standard deviations of the experimental data for the test suite with 5 specific experimental tests - this has been clarified. See also Supplementary Figure S8 and the corresponding legend.

- **line 223 simulates → reproduces**. This has been corrected and now reads "reproduces".See p. 17, l. 299.

- **lines 243 – 247 I don't understand that paragraph** The relevant paragraph has been rewritten; see page 19 l. 319 - 323

- **line 255 mechanistic basis?** This has been rewritten for clarity; see p. 20.

- **line 279 Our model has so far shown that 10 Hz rMS produces LTP concentrated in proximal dendritic locations. → elicits (...) localized in proximal dendrites** This has been rewritten and now reads "elicits" and "localized", see l. 310
- **line 299 probably an effect of tonic inhibition.** See Supplementary Figure S4 for an exploration of the effects of tonic inhibition.

Reviewer 2

- **Reviewer 2 major issue 1: The model is not properly described. I would suggest including at least a minimal description of all model components (especially the ones underlying the parameters listed in the tables) either in Methods or Supplementary material. The online model entry has issues too. I tried to use the model they uploaded in dropbox but faced issues. The function "t2n_initModelfolders" that is used by the simulation scripts is not defined anywhere. Also the scripts point to a folder ../Models which doesn't exist. The README file could be more informative - it tells me about the I/O needs (which is great) but not about the runtime of the simulations.**

  We have added a comprehensive description of all model components to the Methods section (see p. 5 - 13) and provided improved instructions for how to use the shared code, in the new updated Readme file. The function t2n_initmodelfolders and related code is now explicitly included in the shared code: `https://www.dropbox.com/scl/fo/31hxt5ckf980u3f7exoqf/ACgS-I-AHtFrM8v75V87OX8?rlkey=x8hyjd1lg8tenrigadrfu1ma st=m3o6ful0&dl=0`

- **Reviewer 2 major issue 2: The terminology related to the stimulus is somewhat confusing - at least if you're not completely familiar with the protocols used in this branch of neuroscience. From the Methods I understand that there are two stimuli: 1) The synaptic activation (Schaffer collateral synapses) and 2) a somatic current injection. In the experiments of Fig. 2, only the synaptic stimulation is mentioned in the main text, but the caption also mentions local electrical stimulation. What is this local electrical stimulation? Later it is termed "local electrical stimulation of hippocampal CA1 pyramidal cell synapses". If this is only synaptic stimulation, I would call it plainly "synaptic stimulation" or "Schaffer collateral stimulation" to distinguish from the somatic stimulation. However, in Fig. 2 I'm left wondering whether the somatic stimulation is in place or not. It is also unclear what is the main difference in the protocols between Fig. 3-4 and Fig.5: why is there no weight change for proximal synapses in Fig.5 while there was a strong potentiation in Fig.3-4?**

  We have explained how the varying stimulation protocols work in the Methods section, specifically the difference between electrical and magnetic stimulation and how these are modelled, see p.12. We followed the suggestions of the reviewer and regarding terminology, we decided to use "synaptic stimulation" or "local electrical Schaffer collateral

stimulation" to refer to electrical stimulation (because classical extracellular stimulation electrodes activate only presynaptic axons whereas magnetic stimulation fires both pre- and postsynaptic neurons). In Fig. 2 somatic stimulation is not in place. We mention it now in the Figure 2 legend. The main difference between Figs 3-4 and the Fig.5 (currently Fig. 6) is that in Fig 6A only distal perforant path synapses in the OLM layer were stimulated and, in Fig. 6A, the postsynaptic cell was stimulated by the magnetic field and at the same time all incoming synapses were stimulated (based on the assumption that magnetic stimulation fires also presynaptic neurons in the slice or slice culture) - and the stimulation pattern was a theta-burst instead of repetitive 10Hz. Therefore, there is only weight change for distal synapses in Fig.6A and because theta-burst depolarizes entire cell, there is a global change for all synapses in Fig.6B (as opposed to 10 Hz magnetic protocol, which mainly triggers bAPs).

- **Reviewer 2 major issue 3: One problem with the terminology is that "rMS" ("repetitive magnetic stimulation") seems to occasionally used as if it was a term for a specific stimulation protocol. Please use another term if you refer to a specific protocol. Magnetic TBS is also repetitive magnetic stimulation. In particular, the manuscript might benefit from a section or table in the Methods where each protocol is explained.**

  We have added explanatory text to the Methods section about the difference between RMS-TBS and periodic RMS and the electrical stimulation protocols used, see p. 12-13.

- **Reviewer 2 major issue 4: Please include one panel where the time course of the synaptic weight change is plotted for one of each synapse class (green, purple magenta) in Fig 3. Would be also useful in Fig. 2. It would also be beneficial for the reader to see panels that show what is the contribution of pre- vs. postsynaptic LTP/LTD to the reported LTP/LTD outcome, similar to Figure 1E in Ebner et al. 2019.**

  We have added a plot showing the time course of pre- and postsynaptic weight change for each of the classes of synapse over the stimulation period - see our new Figure 4. This allows the reader to see also the contribution of pre- vs. postsynaptic LTP/LTD.

Reviewer 2 minor issues:

- **Methods: "To simulate dendritic TTX application, the conductances of all sodium channels in the dendrites of the cell were set to zero": Only dendritic Na+ channels or also somatic/axonal ones? What happens when all Na+ channels are blocked, are the results the same?** We set to zero only dendritic Na+ channels - this was sufficient to replicate data because an additional elimination of Na+ channels in the soma and axon inhibits the ability of the cell to spike at all. Taken together, the results are the same irrespective of dendritic only or global Na+ channel blockade.

- **Methods: "In addition to the rMS pulses, we applied a random asynchronous input at 3 Hz to mimic spontaneous neuronal activity in organotypic slice cultures.": You**

should say this is Poisson-type activity. On line 397 you mention it is "3 Hz background activity with random jitter" - this is also less informative than just saying that it's a 3-Hz Poisson process. Thank you, we have renamed the description of this to 3-Hz Poisson activity. See l. 222, 276, 503.

- **Methods: The TMS waveform generator is not a standard method, at least to me. Please explain in the methods what the 275 V/m means in practice and how it is implemented in the neuron model. A brief pulse of electrical current applied in a distance-dependent manner to each compartment? I do not find anywhere information on how long these pulses are.** We have clarified in Methods (see lines 182-192) that this is not a current injection, it is a simulated magnetic stimulation pulse, which induces an electric field pulse. This is a standard method to simulate magnetic and electric fields (induced by a TMS-coil) using SimNIBs - as employed before by us (Shirinpour et al. Brain Stimulation 2021) and other groups, which are applied to the entire cell membrane (as a change in extracellular potential in NEURON). This depolarizes neuronal membrane leading to an axonal action potential. 275 V/m is a higher intensity than used in vivo. This is a well-known problem of single cell simulations - probably mainly due to the fact that single cells are not embedded in a circuit.
- **Fig. 2: Green colour missing from (A). However, the color coding doesn't seem to be used at all in Fig. 2?** The lack of green color in Fig.2 signifies that only the stratum radiatum and stratum oriens (the colored regions) are targeted, and the use of only green color in Fig. 5A signifies that only the stratum lacunosum-moleculare is targeted.
- **Fig. 2: Are the simulation results (red circles) averages over 128 synapses? A minor point, but I would put the simulations with solid line and experimental data with separate points because you can easily make more simulated results and (supposedly) get a smooth curve while you are using experimental data as given in Ikegaya et al.** The simulation results are an average of all synapses that were directly stimulated by the stimulation protocol (In this case those in the blue and magenta regions). In TMS simulations where we focus on proximal LTP (figures 3 and 4 and supplementary figures S3 S4 and S5 we consider only proximal str. radiatum (closer than 200 um) synapses in the average. This has been clarified in the axis labels of the relevant figures. As for the visualization of the simulation vs. data in Figure 2; thank you for this suggestion; it has been implemented in Figure 2.
- **Fig. 3: What drives the LTD in distal synapses in 10 Hz stimulation? Do the proximal LTP and distal LTD become stronger for increased frequencies?** LTD in distal synapses during 10 Hz stimulation is driven by the synapse experiencing presynaptic spikes without subsequent postsynaptic spikes (i.e., without sufficient backpropagating action potentials (bAPs) and local depolarization). Under these conditions, the LTD pathways are activated due to a low value of the variable "C," which approximates low postsynaptic calcium levels. Because these synapses are located distally, backpropagating action

potentials are attenuated to a level that remains below the threshold required for LTP induction. Regarding frequency dependence, higher stimulation frequencies do enhance LTP, as briefly explored in Supplemental Figure S3.

- **Fig. 4: I would suggest not to call the control experiments ”baseline”, since usually ”baseline” in LTP/LTD terminology means the status of the system before the plasticity-inducing protocols.** We thank the reviewer for the suggestion; we have renamed these experiments ”no perturbation” (see updated Figure 5 on p.22) or ”control” (see Figure 5 legend).

- **Fig. 2 and 4: Do the manipulations (blocking of inhibition, Na+ channels, NMDA currents, L-type Ca2+, or extracellular Ca2+) affect the baseline synaptic conductance?** Blocking NMDA currents does change baseline synaptic conductance, but the other modifications do not. We have clarified this in Results, see p. 21 l. 369 - 372 and Methods, see p. 11, l. 193-195.

- **Discussion: There are two paragraphs about metaplasticity (which was not included in the model), which is a bit difficult to follow. The authors seem to equate metaplasticity modelling with modelling using plasticity rules that are able to take into account events further back in time. While this can be considered an important aspect of metaplasticity, it is not the only one. Whether plasticity occurs depends also on the state of neuromodulation and other internal states that may or may not be affected by the history of synaptic inputs and spiking responses. I would therefore suggest a change in discussion the limitations of the model that you start by discussing the biological phenomena that you cannot yet describe with the model and then suggest the additional components needed to capture them rather than the other way around.** We have reframed the discussion of metaplasticity in these terms, see l. 541-551 - we thank the reviewer for this suggestion.

Reviewer 3

Reviewer 3 minor comments:

- **Please make sure that acronyms are explained before first use – e.g. rMS is line 44.** We have checked acronym use and ensured that they are all defined on their first use.

- **Line 54 “better explain” – better than what?** We have clarified the language here, replaced ”better explain” with ”reproduce”, see l. 68.

- **Line 115: “... model IS shown ...”** Corrected. We thank the reviewer for the suggestion, see l. 165.

- **Line 174. The reference should be S5** Corrected. As we now added new supplementary figures, the reference now refers to S8, see p. 8 l. 134.

- **Line 176 onwards: this first section of Results seems to belong to the Method section.** We see the development of a new modelling framework for biophysically detailed simulations of TMS-induced synaptic plasticity as a methodological result (a creation of a new tool) and would prefer to keep this section in Results. However, if the reviewer still prefers to move it to Methods, we are happy to do so.

- **Moreover, I think it would further increase the accessibility and completeness of the paper, if the authors would, in a bit more detail, describe the mathematical and biophysical foundations of the used neuron model, including the way the induced electric field is coupled to the neuronal states. I understand that these details are given elsewhere, but at least a brief summary with the most important equations would be helpful.** We have implemented this detail in the Methods section, explaining the coupling mechanism between the electric field and the postsynaptic membrane; see lines 197-221.

Nicholas Hananeia
Computer-Based Modelling in the field of 3R Animal Protection, ICAR3R, Faculty of Medicine, Justus Liebig University, Giessen, 35390
