# Supplementary material for: Multi-scale modelling of location- and frequency-dependent synaptic plasticity induced by repetitive magnetic stimulation in the dendrites of pyramidal neurons
Source: PLoS Comput Biol. 2025 Nov 25;21(11):e1012295. doi: 10.1371/journal.pcbi.1012295 (PMC12671772; doi:10.1371/journal.pcbi.1012295)
Supplement: Attachment — Submitted filename: Hananeia_et_al___Rebuttal_letter_R2.pdf [file pcbi.1012295.s010.pdf]

PLoS Computational Biology, November 6, 2025
Cambridge, UK

Dear editors of *PLoS Computational Biology*,

We would like to submit the attached revised manuscript entitled "Multi-scale modelling of location- and frequency-dependent synaptic plasticity induced by repetitive magnetic stimulation in the dendrites of pyramidal neurons" for publication as a journal paper in PLoS Computational Biology.
We appreciate the reviewers' comments on the remaining minor issues left in the manuscript and we hope that with these changes, it is ready for publication. We have implemented all of the minor changes suggested in the text and figures, and we have improved the accessibility of the code, including providing an example fully generated version. We provide a detailed response to the reviewers' individual comments below.

We again thank the reviewers for their time and effort, given how long that this paper has been in revision.

We hope that the revised manuscript will be acceptable for publication in PLoS Computational Biology.

We look forward to hearing from you,

Nicholas Hananeia, Christian Ebner, Christos Galanis, Hermann Cuntz, Alexander Opitz, Andreas Vlachos & Peter Jedlicka

Reviewer 1

The manuscript was thoroughly rewritten and is a pleasure to read (and look at!).

Minor comments:

- **97 is ->are** Text now reads are - channels, see line 96-98
- **probably all ion names should be in mathrm mode** Change has been made; all ion names are in mathrm mode, see lines 96-100.
- **104 - 106 was modified ->consisted of** Text now reads "consisted of", see line 103
- **107 - what is MS?** Text now reads "magnetic stimulation", see line 105-106
- **190-191 - what about opening of L-type Ca channels in the spines due to the depolarization accompanying plasticity induction?** We describe the blockade of L-type Ca channels on line 182-184 in Methods and line 376-381 in Results. Blockade of these channels reduced LTP. As for spines, we did not explicitly model spine compartments in our reduced morphology models, but we expect that simulating the blockade of L-type Ca channels in explicit spines would produce similar results.
- **Fig 2 panel B "Inhibition enabled" ->maybe "Control"** Figure 2B Panel title now reads "Control".
- **Fig 2 panel C "Inhibition disabled" ->maybe "Blocked inhibition"** Figure 2C Panel title now reads "Blocked inhibition"
- **I would probably change Simulation to Model** Figure 2 legends now read "Model" rather than "Simulation".
- **286 strongest fit ->best fit** Text now reads "best fit", see line 286
- **287 weakest fit ->worst fit** Text now reads "worst fit", see line 287
- **292 this ->a** Text now reads "a", see line 292
- **314 What is the LTP measure? It needs to be defined.** Text now explains what LTP measure is, see line 314
- **Fig 3 panel B: Lenz et al (2015) is looking at aEPSC amplitude, it might be a good idea to compare EPSC amplitude instead of approx. weight.** We clarify that we are directly measuring weights before and after; we considered relative aEPSC amplitude as an experimental proxy for weight value, and used direct measurement of relative weight change in our simulations for ease of measurement in large simulations with many synapses. It is worth noting that the proximal dendritic location EPSCs in our model are on the order of 5pA when measured at the soma, which is in order to the range seen in Lenz (2014) figure 2B.

- **Fig 3 caption decreasing with lower frequency ->decreasing with decreasing stimulation frequency** Fig.3 caption now reads ”higher stimulation frequency” and ”lower stimulation frequency”
- **319 remove word ”here”** ”Here” has been removed, see line 320
- **320 classical electrical presynaptic stimulation ->commonly used presynaptic plasticity-inducing stimulation protocols** Text now reads commonly ”used presynaptic plasticity-inducing stimulation protocols”, see line 321-322
- **321 localized to ->limited to** Text now reads ”limited to”, see line 324
- **321 the proximal dendrites ->proximal dendrites** Text now reads ”proximal dendrites”, see line 324-325
- **325 In all cases ->For all dendritic locations** Text now reads ”For all dendritic locations”, see line 327
- **327 we experimented with - please rewrite** Text now reads ”To determine what the effect of higher frequencies on magnetic-stimulation induced LTP was, we implemented similar 900-pulse protocols at 15 and 20 Hz.”, see line 330-331
- **333-336 I do not understand this sentence, please rewrite** Text has been rewritten for clarity, see lines 335-337.
- **338 depolarisation of neurons ->maybe excitability?** Text now reads ”excitability”, see line 342-343
- **373 something is missing in this sentence, plasticity induction maybe?** Phrase ”plasticity induction” added in rephrase, see line 379
- **377 I think simulated is unnecessary** ”Simulated” has been removed, see line 381
- **379 amplitudes of what?** Text now reads ”weight changes” rather than ”amplitudes”, see line 383
- **Fig 5 panels (C-D) no perturbation ->control** Figure 5 legends now read ”Control” instead of ”No perturbation”
- **panel C NMDA ->NMDAR** Figure 5 Panel C legend now reads ”NMDAR” instead of ”NMDA”
- **panel D L-Type Ca2+ suppressed ->L-type VGCC, add VGCC definition in the caption** Figure 5 panel D legend now reads ”L-type VGCC suppressed” and definition of VGCC provided in Figure 5 caption
- **401 ->Nice!**

- **404 ->rewrite sentence** Sentence has been rewritten for clarity, see line 408-410
- **411 electrical TBS ->maybe electrically induced TBS** Sentence has been rewritten to say ”electrically induced TBS”, see line 416
- **419 stimulation is missing from the sentence** ”Stimulation” has been added to sentence see line 424
- **Fig 6 caption inhibition is disabled ->inhibition is blocked** Now reads as ”inhibition is blocked”, see Figure 6 caption
- **Fig 7 deflections – depolarizations** Now reads ”depolarisations”, see fig 7 caption
- **472 specific changes of synapse ->specific changes of synaptic strength** Now reads ”synaptic strength”, see line 477
- **475 very simple synaptic modelling ->included a very simplistic model of synaptic activation (?)** Text now reads ”included a very simple model of synaptic activation”, see line 480
- **502 stimulus ->stimulation** Text now reads ”stimulation”, see line 507

Reviewer 2

The authors have revised the manuscript and it's almost ready for publishing in my opinion. The only issues I have left are related to the publication of the code and reproducibility.

- **I agree with Reviewer 1 that the work suffers from being coded in MATLAB. Just to make this revision, I had to re-initiate my MATLAB license, adding a cost to my university's software budget. The code is also using toolboxes (randsample.m), which adds additional costs. I know it's comfortable to use the software one has become acquainted with, but a longer term solution would be for the authors to start using Python in their future publications.** Unfortunately, this project is built atop a legacy codebase that has integrated multiple MATLAB-exclusive tools (Specifically TREES Toolbox and T2N).

  We have considered this criticism in our ongoing work, however. We have started translated the TREES Toolbox from MATLAB into Python. Unfortunately, finishing this is beyond the scope of the current manuscript.
- **And please don't use white spaces in folder names.** We have modified folder names to not use whitespaces.
- **The code is now made more readable with new README files compared to the first version. Sadly, I have to conclude that even after many approaches I cannot run the simulations on my Mac. It seems that the system is tuned for Windows, and it would**

require large modifications to make it runnable in Mac, and perhaps the same for Linux/Unix systems. When preparing the final code publication, I would suggest that the authors 1) try it on Linux/Unix/Mac machines, and provide information how to make it runnable there, or 2) provide a "default" hoc file that contains one typical simulation and is runnable without all the MATLAB code generation. With the hoc and mod (and morphology files) in place, anyone can run the model regardless of their operating system and regardless whether they have MATLAB installed or not. Then the work would be at least minimally reproducible for all. There are also options to save large simulation scripts into online databases other than Github and ModelDB. Currently, it seems to me that the crucial hoc files are being made by the MATLAB script, and thus if that script does not work I cannot run any simulations. We have uploaded a ready-to-execute version of the simulation to Zenodo, which can be run without MATLAB. We describe the format of the output data in the README file so that it can be visualized without using our MATLAB visualization scripts. We have tested this ready-to-execute version on both Windows and Linux.

- **Mostly the synaptic changes in stratum radiatum are discussed, while the changes in stratum oriens are predicted to be larger (1.5x to 2.0x in stratum oriens compared to 1.1x to 1.5x in stratum radiatum in Fig. 3). Could you relate these predictions to some experimental data?**

  We modelled the only data that were available for RMS-initiated LTP induction in the hippocampus; specifically Lenz et al. (2015) This remains a prediction because we only have data from the proximal, middle and distal synapses in the stratum radiatum (see Lenz et al.(2015) Fig. 2 and Fig. 3) and unfortunately we are not aware of any data showing similar results in the stratum oriens, so this is a prediction that should be tested experimentally.

- **Fig. 4: Use seconds or minutes on x axis instead of ms*1e4** Updated. Figure 4 time axis is now displayed in seconds.

- **L.344 "Importantnly"** Typo has been corrected, see line 348

- **L.431: "experiments by Kim et al. showed that distal LTP is dependent on sodium dendritic spikes [48]". [48] seems to have two different protocols: strong, local TTX close to soma/proximal dendrites, and bath application of low concentration of TTX that effectively blocks the dendritic Nav channels. I suppose the authors model the latter. However, I would assume that also the somatic Nav channels are somewhat affected by this procedure - there is no mentioning of specifically targeting the dendrite in the bath application. In [48] they suggest that bath application of low-concentration of TTX effectively blocks the dendritic Nav channels because they are low in number, while the Nav channels in the soma are less affected due to their density. This discrepancy should be discussed in the Discussion - alternatively, you could show that a**

simultaneous partial blockade of somatic Nav channels gives reasonable output.

We ran the suggested simulations of blocking dendritic but also partially somatic Na+ channels and the result is near-identical to the case we initially simulated. We describe it now in the revised Figure 8 caption.

Reviewer 3

My (relatively minor) concerns from the previous review have been adressed satisfactorily. I endorse publication. We thank Reviewer 3 for the endorsement for publication.

Nicholas Hananeia
Computer-Based Modelling in the field of 3R Animal Protection, ICAR3R, Faculty of Medicine, Justus Liebig University, Giessen, 35390
